# *Clostridium difficile* in Asia: Opportunities for One Health Management

**DOI:** 10.3390/tropicalmed4010007

**Published:** 2018-12-28

**Authors:** Deirdre A. Collins, Thomas V. Riley

**Affiliations:** 1 School of Medical & Health Sciences, Edith Cowan University, Joondalup 6027, Australia; deirdre.collins@ecu.edu.au; 2 Department of Microbiology, PathWest Laboratory Medicine, Nedlands 6009, Australia; 3 School of Veterinary & Life Sciences, Murdoch University, Murdoch 6150, Australia

**Keywords:** *Clostridium difficile*, Asia, epidemiology, One Health

## Abstract

*Clostridium difficile* is a ubiquitous spore-forming bacterium which causes toxin-mediated diarrhoea and colitis in people whose gut microflora has been depleted by antimicrobial use, so it is a predominantly healthcare-associated disease. However, there are many One Health implications to *C. difficile*, given high colonisation rates in food production animals, contamination of outdoor environments by use of contaminated animal manure, increasing incidence of community-associated *C. difficile* infection (CDI), and demonstration of clonal groups of *C. difficile* shared between human clinical cases and food animals. In Asia, the epidemiology of CDI is not well understood given poor testing practices in many countries. The growing middle-class populations of Asia are presenting increasing demands for meat, thus production farming, particularly of pigs, chicken and cattle, is rapidly expanding in Asian countries. Few reports on *C. difficile* colonisation among production animals in Asia exist, but those that do show high prevalence rates, and possible importation of European strains of *C. difficile* like ribotype 078. This review summarises our current understanding of the One Health aspects of the epidemiology of CDI in Asia.

## 1. Introduction

*Clostridium difficile* is a ubiquitous spore-forming anaerobic bacterium which colonises the infant mammalian and avian gastrointestinal tract before the gut microflora has been established [1]. This “virgin” gut environment is replicated in mammals of all ages during and after antimicrobial exposure, or because of other circumstances that deplete or change the gut microflora. While human infants may not yet express the receptor for *C. difficile* toxins [2], older children and adults who become infected with toxigenic *C. difficile* can experience toxin-mediated disease ranging from self-limiting diarrhoea to life-threatening pseudomembranous colitis (PMC) and/or toxic megacolon.

*C. difficile* infection (CDI) has been predominantly a healthcare-associated illness, with the majority of cases being of advanced age, with comorbidities and a history of recent hospitalisation or treatment for illness. Increasing reported incidence rates in many regions [3] can partly be explained by the adoption of highly sensitive PCR testing [4] over the past decade, however, rates of community-associated (CA)-CDI are also rising [5,6]. While *C. difficile* spores can survive for long periods of time in healthcare environments due to their resistance to many disinfectants, recent advances in whole genome sequencing (WGS) studies have shown that up to 50% of CDI cases may be acquired from sources outside of healthcare facilities [7], implying environmental exposure accounts for a considerable proportion of CDI cases. High rates of *C. difficile* colonisation among food production livestock in which antimicrobials are frequently overused [8] have increased the risk of zoonotic transmission of *C. difficile* to humans [1]. Studies show high prevalence of *C. difficile* contamination of outdoor environments [9,10] and root vegetables [11] due to the use of contaminated animal manure as fertiliser. WGS has identified clonal groups of *C. difficile* isolated from both humans and animals [12], further supporting the possibility of zoonotic transmission of *C. difficile* from production animals to humans.

Intercontinental epidemics of CDI demonstrate the potential for international spread of *C. difficile*. Examples include the severe outbreaks in North America and Europe caused by clonal strains of ribotype (RT) 027 *C. difficile* originating in North America [13], and outbreaks of clindamycin-resistant strains of RT 017 across Asia, Europe and North America [14,15,16,17]. CDI epidemiology has been well documented in North America, Europe and, to a lesser extent, in Australia [5,6,18,19]. Different molecular types of *C. difficile* circulate in these respective regions, primarily ribotype (RT) 027 in North America and, until recently, Europe [20,21], and RT 014/020 in Australia [22]. To date, CDI has been largely under-diagnosed, under-reported and under-investigated in Asia, despite being home to 60% of the world’s population, due to poor awareness among physicians and often inappropriate testing [23]. 

Over recent decades, growing economies and expanding populations across Asia have led to a rising middle class and ageing population with increasing demands for medical and aged care facilities. This wealth increase has also led to a greater appetite for meat and meat products, which has triggered a massive increase in meat consumption [24] and huge population expansion among meat production livestock, most notably pigs, chicken and cattle. This large-scale production farming, growing populations accessing healthcare facilities and widespread overuse of antimicrobials [25] make Asia an environment which is highly conducive to transmission of *C. difficile*, among both humans and animals. 

The One Health paradigm approaches public health from a collaborative, multi-sectorial point of view, aiming to integrate policies, legislation and research to achieve better public health outcomes. It is particularly relevant to biosecurity, encompassing zoonotic infection, the rise of antimicrobial resistance and food safety. Given widespread colonisation of production animals and environmental contamination with *C. difficile* spores, management and control of CDI should use a One Health-based approach. This review examines our current knowledge of *C. difficile* in Asia from a One Health perspective.

## 2. Epidemiology of CDI in Asia

### 2.1. Diagnostic Practices in Asia

The prevalence and incidence rates of CDI can vary widely according to the testing method used. Diagnostic assays range from enzyme immunoassays (EIAs) detecting glutamate dehydrogenase (GDH) and/or toxin (A, B or both) to PCR for the *tcdA* or *tcdB* genes, to traditional culture and cell culture cytotoxicity assay (CCCA). No diagnostic test besides CCCA is suitable as a stand-alone test since toxin EIAs have low sensitivity, and PCR, GDH EIA and culture cannot rule out cases of transient colonisation [26]. CCCA is laborious and time-consuming so it is not routinely employed in diagnostic settings. Reports from Asia have indicated inappropriate testing in the past, particularly use of toxin A EIAs, which will underdiagnose CDI in Asia due to the high prevalence of toxin A-negative/toxin B-positive (A-B+) RT 017 and RT 369 strains [23]. According to a systematic review of studies in Asia, the most commonly performed tests were culture (71%) followed by EIA (52%) and PCR (51%) [27].

### 2.2. Estimated Prevalence and Incidence of CDI in Asia

Culture and PCR identify toxigenic *C. difficile* at high prevalence ranging from 9%–11% [28,29,30] in South-East Asia, while toxin EIA was positive in only 3%–5% of the same study specimens [28,29]. A systematic review of studies of CDI from Asia found a mean overall prevalence of 14.8% among hospital inpatients and outpatients, varying significantly from 2.0% to 61.4% across studies, and 16.4% among hospitalised patients with diarrhoea. The pooled incidence rate of CDI in Asia was calculated by meta-analysis at 5.3/10,000 patient days (95% CI 4.0–6.7) [27]. The random effects pooled CDI-related death rate was estimated at 8.9% (95% CI 5.4%–12.3%) by meta-analysis of existing studies [27], while a 13-country descriptive study with 600 recruited CDI cases found a lower mortality rate of 5.2% [31].

Studies in Singapore have demonstrated how changing testing practices have affected incidence rates. The incidence of CDI in Singapore was reported as increasing during the early 2000s, and from 2001 to 2006 the number of samples tested each year increased from 906 to 3508, with the percentage of positive samples increasing from 7% to 11% over the same period [32]. Subsequently, the incidence rate appeared to reduce, which was due to continuing increases in the number of samples being tested (4348 in 2006 to 6738 in 2008 between two hospitals) [33]. This suggests that increasing awareness and vigilance among physicians for possible cases of CDI led to more extensive testing among patients with diarrhoeal disease. Limited resources in some settings have resulted in still inadequate or no testing for CDI. For example, in a study in the Philippines, patients with CDI were frequently misdiagnosed with amoebiasis according to endoscopic detection of colitis [34]. 

### 2.3. Burden of CDI in Asia

Despite a high prevalence of *C. difficile* in Asia [27,28,30,35], reports of severe outcomes of CDI are rare. Few reports of PMC and toxic megacolon exist from Asian countries [36,37,38,39,40,41,42,43], suggesting they may be less commonly seen than in other regions. Where reports do appear, they are frequently associated with infection with A-B+ strains [36,40]. Recurrence rates are also lower at 9%–13% [31,44,45,46] than those reported from North America (15%–20%) [6] and Europe (16%–22%) [19,47], however definitions of recurrence can vary from 8 weeks to 90 days for reappearance of symptoms after resolution of disease. The apparent rarity of severe outcomes of CDI in the region, such as PMC or toxic megacolon, is likely influenced by the poor awareness of CDI among physicians. As demonstrated in the study in the Philippines, CDI is misdiagnosed as amoebiasis and treated with metronidazole which is often sufficient for resolution of milder cases of CDI, resulting in missed cases [34]. 

### 2.4. Molecular Epidemiology of CDI in Asia 

#### 2.4.1. A-B+ C. *difficile* Strains

The most commonly used molecular typing methods for *C. difficile* are PCR ribotyping and multi-locus sequence typing (MLST). Phylogenetic analyses based on MLST describe at least five major population clades of *C. difficile* [48]. As mentioned before, RT 017/ST37, a clade 4 strain [49], is A-B+ [48] and the predominant strain identified in Asia [23,27,28,35,50]. In China, Korea, Indonesia and Malaysia, RT 017 is generally the most common *C. difficile* strain in circulation, and it is also prevalent in Japan (referred to in older papers as ribotype “fr”), Taiwan, Hong Kong, Thailand and Singapore [28,29,30,51,52,53,54,55,56]. Exposure to antineoplastic agents, use of nasal feeding tubes and care in one particular hospital ward were associated with infection with RT 017 strains in a hospital in Japan [57]. *C. difficile* RT 017 has also caused major outbreaks of CDI outside of Asia, in Canada [58] and Europe [15,16], and is frequently reported as having enhanced fluoroquinolone and clindamycin resistance [15,16], a feature that has most likely contributed to its success as an epidemic strain.

The emergence of *C. difficile* RT 369/ST81, another clade 4 A-B+ strain, is also of interest and warrants close monitoring [31,59,60]. This strain apparently emerged first in Japan, where historically it was referred to in the literature using local nomenclature as “trf” [60,61]. It appears that RT 369 caused outbreaks of CDI in hospitals in 2000 and 2001, when ribotyping was not performed [57,60,62]. The first report of RT 369 was in a study conducted on isolates collected from outbreak and non-outbreak situations from 2009–2013 in Japan. This study detected RT 369 in an outbreak setting in a hospital in 2009 [60], and it is now one of the most common strains in circulation there [31,59]. RT 369 has since been reported in studies from China as the cause of a nosocomial outbreak among hospital patients in Shanghai in 2014 and 2015 where it was the most common strain in circulation. RT 369/ST81 strains are also reported to have higher rates of resistance to clindamycin, ciprofloxacin and moxifloxacin compared with other strains, and a higher sporulation rate than RT 017/ST37 strains [63,64]. 

#### 2.4.2. Binary Toxin-Positive *C. difficile* Strains

Many but not all binary toxin-positive (CDT+) *C. difficile* strains tend to group in phylogenetic clades 2 and 5, and have been associated with epidemics of CDI in North America (RT 027/ST1, clade 2) [13,65], Europe (RT 078/ST11, clade 5, and RT 027/ST1) [19,21] and Australia (RT 244/ST41, clade 2) [66] in recent times. In contrast, CDT+ strains have been only sporadically reported from Asia and major epidemics like those seen elsewhere have not occurred [67]. Most cases of RT 027 infection to date have been reported from China, where 11 cases were reported from one hospital over 3 years [68]. RT 027 also caused CDI among seven patients across four hospitals in Seoul and Gyenngi province in Korea [69], and may be increasing in prevalence in Taiwan, where it was never reported prior to 2015 [70,71]. Most Asian RT 027 *C. difficile* strains investigated to date have not been related to either of the two main epidemic RT 027 lineages referred to as FQR1 and FQR2 [13], and many have been reported as fluoroquinolone-susceptible, unlike the epidemic lineages. 

*C. difficile* RT 078 (CDT+) was reported among eight cases of CDI across three hospitals in China, where it was also isolated from environmental surfaces suggesting nosocomial transmission [72]. RT 078-related strains RT 126 and 127 (both ST11) are more common in Taiwan, where they were the most common CDT+ strains reported from Southern Taiwan between 2011 and 2013 [73]. A subsequent nationwide study from 2015–2016 identified RTs 078, 126 and 127 at significant prevalence among 842 toxigenic isolates (1.5%, 3.1% and 2.9%, respectively), mainly confined to two hospitals [70].

#### 2.4.3. A+B+ *C. difficile* Strains

*C. difficile* clade 1 strains that are mainly A+B+ are also frequently reported from Asia. RT 018/ST17 is the predominant clade 1 strain found in the region with the earliest reports coming from Japan (referred to as ribotype “smz”) [23]. A closely related strain, “smz’”/QX 239/ST17 is now also circulating at high prevalence in Japan [59,60]. RT 018 is now the most common *C. difficile* strain reported from Korea, where it has largely replaced RT 017 [23,74]. RT 012/ST54 and RT 046/ST35 localise to China in particular [75,76,77,78,79], RT 014/020/ST2/14 is widespread across the continent [31], and RT 002/ST8 is most frequently reported from Taiwan and Hong Kong [31,80]. 

#### 2.4.4. Non-Toxigenic *C. difficile* Strains

A notable aspect of the molecular epidemiology of *C. difficile* in Asia is the high prevalence of non-toxigenic strains, particularly in South-East Asia. In recent studies in Thailand, Indonesia and Malaysia [28,30,35], non-toxigenic strains of *C. difficile*, most commonly RTs 009 and 010, QX 083, QX 002 and QX 083, were isolated at a prevalence of 50% among all study isolates. Further north in Asia, non-toxigenic strains are reported less frequently (24%, Taiwan [70] 8%–11%, China [76,79,81]), however, this may be a reflection of the use of diagnostic methods other than culture, which would not detect non-toxigenic strains. These strains are incapable of causing CDI but can colonise the gut when the normal flora are disrupted due to antimicrobial use. Many group in the predominantly non-toxigenic MLST clade 4 [49]. The high prevalence of RT 017 and non-toxigenic strains [28,30,35] suggests that clade 4 may have evolved in the Asian region, but further studies on non-toxigenic strains both in Asia and elsewhere are required to determine whether this is the case.

The unique molecular epidemiology of *C. difficile* in Asia (described in more detail in Collins et al. [23]), particularly the high prevalence of non-toxigenic strains, likely plays a role in the overall apparently less severe manifestations of disease seen in the region. Therapeutic administration of non-toxigenic *C. difficile* can protect against recurrent CDI [82], which occurs more rarely among Asian patients (9.1% of cases) than elsewhere [31]. Thus, it is highly plausible that the high prevalence of non-toxigenic strains is protective against recurrence and possibly reduces risk of exposure to virulent strains in Asia. However, many non-toxigenic Asian *C. difficile* strains are resistant to multiple antimicrobials, possibly due to inappropriate antimicrobial use in the region, and they may pose a risk in terms of transmission of antimicrobial resistance (AMR) genes. There have been concerning, albeit rare, reports of metronidazole-resistant non-toxigenic strains [79,83], which should be closely monitored in the region.

## 3. Prevalence and Molecular Epidemiology of *C. difficile* among Production Animals in Asia

### 3.1. Prevalence of C. difficile Colonisation and Strain Types in Asian Production Animals

While there are few reports on *C. difficile* in animals in Asia, the prevalence appears to be high among production swine across the continent. A study of 120 neonatal piglets in Japan found a prevalence of *C. difficile* of 57.5%; 61.0% of strains were toxigenic [84]. A high prevalence of 19.3% among 910 pigs of all ages across 47 farms has been reported in Korea, with peak prevalence in diarrheic suckling piglets (53.6%) followed by diarrheic sows (40.0%); again, the majority of isolates (86.9%) was toxigenic [85]. In Taiwan, the prevalence of *C. difficile* among 204 pigs on 13 commercial farms was 49% [86]. The only report to date of *C. difficile* among production animals in South-East Asia comes from Thailand, where the prevalence of *C. difficile* was 35% among piglets (*n* = 165), with all 58 isolates reported as non-toxigenic [87]. RT 078 and closely-related strains including RTs 126 and 127 are the most commonly reported toxigenic strains in pigs in Korea (RT 078 86.5%, RT 126 13.5% of toxigenic strains) [85], Taiwan (RT 078 18%, RT 126 28%, RT 127 43% of toxigenic strains) [86] and Japan (RT 078 third most common strain; 19.7% of toxigenic strains) [84], countries where demand for pork and pork products has surged in recent decades. 

### 3.2. Possible International Sources of C. difficile among Asian Production Animals

To date, *C. difficile* RT 078 and related strains RT 126 and 127 have rarely infected humans in Asia apart from in Taiwan [70,73,88] and, given the apparent endemicity of RT 078 among production animals and human infections in mainland Europe and North America, it is plausible that the strain was introduced into northern Asia via live animal imports. Supporting evidence has been reported from Japan; multi-locus variable number tandem repeat analysis (MLVA) found that Japanese piglet isolates clustered with European human and pig RT 078 strains, giving a strong likelihood that they were imported into Japan from Europe via live breeding pig imports [84]. Live breeding pigs and cattle are imported from Europe, Australia and North America to many Asian countries including Japan [89], China, Taiwan, Vietnam, Cambodia, Malaysia and Thailand (ahdb.org.uk). RTs 078 and 127 are common among cattle and pigs in Europe [90] and RTs 126 and 127 are frequently reported in cattle in Australia [91].

*C. difficile* RT 078 has also been reported in thoroughbred racehorses, which are frequently traded internationally, in Japan. Five cases of postoperative colitis were documented from the same facility, indicating contamination with a single clone [92]. Further analysis using WGS of RT 078 strains from Japanese racehorses identified a sub-lineage associated with a nosocomial outbreak. RT 027 and RT 017 were also reported, with high relatedness to several reported European strains including clinical isolates from Ireland [93], a prolific producer of racehorses.

## 4. Discussion

### 4.1. Systematic Testing Is Required to Identify True CDI Cases in Asia 

Introduction of systematic, comprehensive testing for CDI across Asia could provide a better understanding of the epidemiology of CDI in the region, particularly accurate measurement of incidence and prevalence, and deepen our understanding of the burden of CDI. While there is still considerable international debate about optimal testing practices for CDI, colonisation rates with both toxigenic and non-toxigenic *C. difficile* among hospital inpatients are particularly high in South-East Asia. Many Asian countries are popular destinations for “medical tourism” and there is a risk of transmission of strains via medical tourists returning to their own countries after their treatment. Due to the high prevalence of colonization, it is important to use a diagnostic test which will discriminate true cases of CDI from cases of colonization. GDH and toxin EIA can be performed at relatively low cost and will identify most cases of true infection, despite its lower sensitivity, so it may be the best choice currently for Asian laboratories in developing countries. 

Given the apparently uniquely high prevalence of non-toxigenic *C. difficile* strains in Asia, particularly in South-East Asia, it is important to monitor colonization as well. The high prevalence suggests that hospital environments may be heavily contaminated due to poor cleaning or hand hygiene, which puts vulnerable patients at higher risk of CDI. Monitoring of *C. difficile* colonization would also allow further investigation of whether non-toxigenic *C. difficile* colonization is protecting Asian patients from developing CDI and reducing their risk of recurrent disease. 

### 4.2. One Health Implications of CDI in Asia

#### 4.2.1. *C. difficile* in Asian Production Animals Warrants Close Observation 

While there are still relatively few reports of *C. difficile* among Asian production animals, and no reports yet of environmental contamination, the prevalence of *C. difficile* among pigs across Asia is markedly high. Given the significantly increasing demands for pork and pork products, particularly in China and Taiwan, biosecurity measures to ensure these meat products do not pose a threat to humans should include monitoring for *C. difficile* contamination. A spatial epidemiology study in the USA identified increased risk of CA-CDI among people living close to livestock farms [94]. China currently holds half the world’s pig population in addition to being the most populated country in the world, so there is a significant risk of infection of a substantial population. In Taiwan, the presence of “hypervirulent” RT 078 and related strains among pigs and increasing prevalence of these strains among clinical cases of CDI suggests transmission of strains between pigs and humans has already occurred. This could be confirmed using WGS studies, as described in an Australian study showing clonal relationships between *C. difficile* isolates from human clinical cases and pigs located thousands of kilometres apart [12].

#### 4.2.2. Live Animal Imports and Exports: Plausible International Routes of Transmission of *C. difficile*

Genotypic studies of pig and racehorse *C. difficile* isolates from Japan are showing a possibly significant international transmission route of *C. difficile* via live animal imports and exports. The international live animal trade market is a growing sector. From a One Health perspective, it is most important to monitor animals traded with the intention of farming for meat production, as these are kept in close quarters and are thus frequently prophylactically treated with antimicrobials to reduce risk of infection and loss of stock.

## 5. Conclusions

A One Health approach will be important in management and control of CDI in Asia. It is most important to establish comprehensive testing policies, to identify the true incidence of CDI in Asia before being able to implement effective control measures. 
